# Stress load during childhood affects psychopathology in psychiatric patients

**DOI:** 10.1186/1471-244X-8-63

**Published:** 2008-07-23

**Authors:** Katja Weber, Brigitte Rockstroh, Jens Borgelt, Barbara Awiszus, Tzvetan Popov, Klaus Hoffmann, Klaus Schonauer, Hans Watzl, Karl Pröpster

**Affiliations:** 1 Department of Psychology, University of Konstanz, Germany; 2 Center for Psychiatry Reichenau, Germany

## Abstract

**Background:**

Childhood stress and trauma have been related to adult psychopathology in different psychiatric disorders. The present study aimed at verifying this relationship for stressful experiences during developmental periods by screening stress load across life in adult psychiatric inpatients with different diagnoses compared to healthy subjects. In addition, a relationship between the amount of adverse experiences and the severity of pathology, which has been described as a 'building block' effect in posttraumatic stress disorder (PTSD), was explored for non-traumatic events in psychiatric disorders other than PTSD.

**Methods:**

96 patients with diagnoses of Major Depressive Disorder (MDD), schizophrenia, drug addiction, or personality disorders (PD) and 31 subjects without psychiatric diagnosis were screened for adverse experiences in childhood (before the age of six years), before onset of puberty, and in adulthood using the Early Trauma Inventory and the Posttraumatic Stress Diagnostic Scale. Effects of stress load on psychopathology were examined for affective symptoms, PTSD, and severity of illness by regression analyses and comparison of subgroups with high and low stress load.

**Results:**

High stress load in childhood and before puberty, but not in adulthood, was related to negative affect in all participants. In patients, high stress load was related to depressive and posttraumatic symptoms, severity of disorder, and the diagnoses of MDD and PD.

**Conclusion:**

Results support the hypothesis of stress-sensitive periods during development, which may interact with genetic and other vulnerability factors in their influence on the progress of psychiatric disorders. A 'dose' effect of stress load on the severity of psychopathology is not restricted to the relationship between traumata and PTSD.

## Background

A burgeoning number of studies point to the influence of adverse or traumatic experiences during childhood on adult psychopathology [1-7]. This influence has been linked to the particular sensitivity of the developing brain and hormonal system in childhood [8]. A higher than normal childhood stress load has been reported for different psychiatric disorders, like depressive disorders [9,10], schizophrenia [11-15], anxiety disorders including posttraumatic stress disorder (PTSD) [7,14,16,17], personality disorders [18,19], and substance abuse [20,21]. Additive or interacting effects of adverse early experiences and subsequent stress have been discussed in the evolution of psychiatric disorder: For instance, a cumulative effect was derived from more severe brain alterations in animals, which had experienced pre-weaning maternal separation plus later exposure to an open elevated platform [22].

Several mediating factors have been discussed to explain the relationship between early life stress and adult psychopathology: (1) As stated above, stress may influence functional and structural systems in the developing brain, including neuroendocrine systems, thereby increasing stress sensitivity [8,23]. (2) Stress alters affect and emotional responding: Pole and coworkers [24] screened 90 individuals without psychiatric diagnoses and found low positive emotion and larger autonomic responses to threatening experimental stimuli in those 25 subjects who reported childhood trauma. Similarly, Cohen and coworkers [25] found a relationship between adverse childhood events and depression and anxiety in over 1500 adults without psychiatric diagnoses. Animal studies have described a behavioral state of 'despair' or 'helplessness' consequent upon prenatal stress or lasting inescapable stressors [26,27] that is related to neuroendocrine alterations [28]. For humans, Lang [29] related distress and negative affect to diminished activity of the defense system in anxious and depressive patients and emphasized that the defense and the reward system overlap with the stress system. In addition to affect, early life stress may influence cognitive end executive functions, thereby contributing to disorder-specific symptoms: Lysaker and colleagues [30] found higher levels of emotional discomfort, but also more pronounced positive symptoms such as hallucinations in schizophrenia and schizoaffective patients with childhood sexual abuse (see also [31,32], poorer performance on executive function tests and work function. (3) Stress may add to other vulnerability factors by reducing coping capacity, which may interact with the progress of psychopathology [33-35]. (4) A 'dose' or 'building block' effect has been reported for PTSD [36] and schizophrenia [32,37], indicating that an increasing number of traumatic experiences increase the risk for developing a PTSD, the severity of posttraumatic or psychotic symptoms and comorbid disorders.

Many studies investigating effects of early life stress focused on distinct disorders and differed in trauma-screening methods. Studies comparing childhood trauma effects between disorders [16,17] found disorder-specific relationships, but also relationships between abuse and specific symptoms like hallucinations across diagnostic boundaries [37,38]. Thus, conclusions regarding a 'dose' effect across diagnostic groups are difficult to evaluate on the basis of the literature. Therefore, the present study explored whether a relationship between early life stress and adult psychopathology can be found irrespective of the specific disorder, whether a subgroup of individuals with high early life stress load can be described across diagnoses, or whether stress load and its relationship with psychopathology varies between diagnostic groups, which would point to a more complex interaction between vulnerability factors.

With this goal, number, type, and frequency of adverse experiences were screened in psychiatric inpatients with different diagnoses for three periods of life: early childhood (before the age of six), the lifespan before the individual onset of puberty, and adulthood (between puberty and current age). From the evidence cited above, we hypothesized (a) a higher stress load in psychiatric patients than in non-psychiatric comparison subjects, (b) a relationship between the amount of stress load experienced early in life (before puberty or even earlier) and the severity of psychopathology in patients, and (c) a similar relationship between adverse experiences and psychopathology as has been described between traumatic experiences and PTSD symptoms or hallucinations [37,38].

## Methods

### Participants

Altogether 102 inpatients of a local Center for Psychiatry (Zentrum fuer Psychiatrie Reichenau) and 36 individuals without psychiatric diagnoses were engaged in the study. The non-clinical sample was recruited by local advertisements and word-of-mouth recommendation. After the exclusion of six patients and five comparison subjects (3 drop outs (1 patient), insufficient knowledge of the German language (5 patients), subclinical psychopathology (3 comparison subjects)), the sample included 96 inpatients and 31 comparison subjects (see Table 1 for demographic and clinical information).

**Table 1:** Demographic and Clinical Data of the Studied Sample

	**Gender ** **Female/** **male**	**Age ** **M ± SD** **(range)**	**Years of ** **education**	**BDI**^a^	**BPRS**^b^	**Medication**
**Patients ** (N=96)	40/56	36.2 ± 12.2 (18–69)	12.4 ± 2.8 (8–20)	17.9 ± 11.3 (0–46)	50.5 ± 10.6 (25–77)	
**Comparison Ss ** (N = 31)	15/16	40.3 ± 15.6 (19–70)	15.1 ± 2.9 (11–21)	3.3 ± 3.9 (0–12)	-	-

**Group ** **Differences**	Chi^2^(1) = 0.4	t(125) = 1.52, p > .1	t(125) = 4.69*	t(119) = 6.58**		

**Major Depressive ** **Disorder** (N=39)	21/18	42.4 ± 13.7 (18–69)	12.9 ± 2.9 (8–20)	23.8 ± 10.9 (1–46)	49.4 ± 9 (27–65)	None:2, N-Mix:15, AD-mix:3, SSRI/SNRI:16, Natyp:1, Benzo:1, MAO:1
**Schizophrenia ** (N = 32)	10/22	32.6 ± 9.1 (19–50)	12.7 ± 3.2 (8–18)	13.3 ± 9.7 (0–36)	49.1 ± 7.9 (34–69)	AD+N:15, AD:1, Atyp:13, Typ:1, Mix-N:1 TCA:1
**Drug Abuse ** **Drug Abuse **	1/14	33 ± 9 (21–48)	10.9 ± 1.5 (8–13)	10.9 ± 8 (1–28)	59.9 ± 11.7 (36–77)	None:15
**Personality ** **Disorder** (N=10)	8/2	27.9 ± 8.1 (18–43)	11.7 ± 1.4 (9–13)	21.4 ± 10.7 (6–44)	44.7 ± 15.4 (25–71)	None:5, Mix:1, TCA:1
**Group ** **Differences**	Chi2(3) = 17.42*	F(3,92) = 7.46**	F(3,92) = 2.2 n.s.	F(3,90) = 9.23**	F(3,89) = 5.95*	

*Note*. ^a^Beck Depression Inventory.^b^Brief Psychiatric Rating Scale. Statistical comparisons: *: p < .01; **:p < .001

Diagnosed by experienced senior psychiatrists using ICD-10 (International Classification of Diseases [39]) criteria, patients received diagnoses from the categories of major depressive disorders (MDD, F31–33), schizophrenia spectrum (F20, F25), drug addiction (F19, F10), and personality disorders (PD, F60.3, F60.31). Diagnostic subgroups differed in gender distribution (the drug addiction subgroup comprising more male participants than the PD and the MDD subgroups and the schizophrenia subgroup comprising more males than the PD group (see Table 1), and age (MDD patients being older than the other groups, who did not differ), but not in educational level. Severity of disorder was evaluated with the Brief Psychiatric Rating Scale (BPRS [40]), Beck Depression Inventory (BDI [41]), and the General Assessment of Functioning Scale (GAF [42]). Drug addicts exhibited higher scores on BPRS and GAF (F(3,89) = 25.51, *p *< .001) than the other diagnostic subgroups, who did not differ. BDI scores were higher in patients with MDD compared to drug abuse and schizophrenia patients. Except for drug addicts, most patients were on medication (see Table 1), the majority receiving combinations either of antidepressants and antipsychotics, typical and atypical antipsychotics, or tricyclic and SSRI antidepressants.

Subjects were only included in the comparison group, if they did not present any sign of a psychological disorder according to the Mini-International-Neuropsychiatric-Interview (MINI [43]) and did not take any psychoactive medication. Comparability with the patient group was confirmed for gender distribution (50 vs. 58% females) and age (p > .1), while groups differed with respect to education: the total years of scholarly education were higher in healthy subjects than in patients (*p *< .001).

### Design and materials

The study protocol was approved by the ethics committee of the University of Konstanz. Participants were informed about the goal of the study and procedures, and signed a written informed consent.

Demographic information was obtained from a standard questionnaire used in the clinical setting, which was extended to smoking habits, drug, and alcohol usage. Adverse experiences or stress load was assessed by the German version of the Early Trauma Inventory (ETI [44]). The interview screens adverse experiences in four domains: general trauma, physical punishment, emotional neglect, and sexual abuse. Any reported experience within each domain is considered as a single event. For each reported event, the age when it started and the age when it terminated are specified, and the event frequency within each year or experience is encoded on a 7-point Likert-scale ranging from 'never within this year' to 'several times a day'. For each year of experience, the frequency ratings were summed up (a) for the time period before the age of six (labeled early life stress, ELS), (b) for the time period before the individual onset of puberty (labeled prepubertal stress, PPS), and (c) for the time between puberty and the current age (labeled adulthood stress, AS). In addition, the number of events was analyzed for each life period. Further measures of stress load were determined with the Posttraumatic Stress Diagnostic Scale (PDS [45]): specific traumatic experiences, a current diagnosis of PTSD, as well as the severity of PTSD symptoms (intrusions, avoidance, hyperarousal). PTSD symptoms were also assessed for the worst non-traumatic ETI event in subjects, who did not report a traumatic event. Finally, prenatal stress was explored using the Prenatal Stress Questionnaire (PSQ [46]). This self-report questionnaire assesses the effects of stress experienced by the client's mother during pregnancy (e.g. disease, accident or loss of partner or close relatives, divorce, etc.), smoking, alcohol, and drug habits, and her psychological and physiological well-being.

Psychopathology was determined with a focus on affective symptoms. In all participants affective symptoms were assessed with the Positive and Negative Affect Scale (PANAS [47]) and the BDI. In patients, psychopathology was further evaluated with the BPRS, the number of hospitalizations, and comorbid drug abuse, and in schizophrenia patients the Positive and Negative Symptom Scale (PANSS [48]).

### Data analyses

Differences in stress load (ETI-scores and number of events) between groups (patients versus comparison subjects and between diagnostic subgroups) were statistically verified by analyses of variance (ANOVA) for the developmental periods ELS, PPS, and AS. Relationships between stress load in the three life periods and psychopathology were evaluated by correlation (Spearman rho, *r*_*s*_) or linear regression analyses and by comparing subgroups of individuals with high and low stress load. For the latter purpose, subjects with ETI-scores exceeding 2 standard deviations of the mean of the comparison group were assigned to a ,high stress' group, while subjects with ETI-scores below the mean of the comparison group were assigned to a ,low stress' group. Significant main effects or interactions were gradually decomposed with follow-up pair wise comparisons corrected with Bonferroni. Statistical significance for all tests was evaluated at the .05 level.

## Results

A significantly higher number of stressful events and higher stress load before puberty was found in psychiatric *patients compared to healthy subjects *(see Table 2 for means, standard deviations, group differences). Complementing the pattern of stress load, traumatic experiences (PDS), and prenatal stress load estimated from the PSQ were significantly higher in patients than in comparison subjects (p < .001; see Table 2).

**Table 2:** Stress Scores for the Different Groups and Periods of Life

	**ELS^a ^** **Events** **Stress load**	**PPS^b ^** **Events** **Stress load**	**AS^c ^** **Events** **Stress load**	**PDS^d ^** **Events** **(Range)**	**PSQ^e ^** **Events** **(Range)**
**Patients **(N = 96)	5.89 ± 5.8	11.01 ± 7.1	5.03 ± 3.6	2.2 ± 1.9	3.7 ± 3.4
	46.4 ± 70.4	172.2 ± 176.7	135.7 ± 155.8	(0–8)	(0–14)
**Comparison Ss **(N = 31)	1.77 ± 2.2	4.39 ± 3.58	4.94 ± 3.1	0.65 ± 0.9	1.6 ± 1.8
	10.7 ± 17.5	45.1 ± 66.0	45.7 ± 49.3	(0–3)	(0–6)

**Group differences:**	14.84, p < .01	24.62, p < .001	F < 1. n.s.	19.14,	11.57,
F(1,125) =	8.15, p < .01	15.29, p < .001	9.99, p < .01	p < .001	p < .001

*Note*. The Early Trauma Inventory (ETI) assesses stress load in four domains: general trauma, physical abuse, emotional neglect, and sexual abuse. Two scores are presented: the cumulated number of experienced events (top rows) and the stress load calculated accordingly to the ETI guidelines (bottom row). ^a^ELS: Early life stress covers stress load in the time period before the age of 6 years. ^b^PPS: Pre-pubertal stress covers stress load in the time period before the individual onset of puberty. ^c^AS: Adulthood stress covers stress load in the time period between puberty and the current age. ^d^PDS: Posttraumatic Stress Diagnostic Scale assesses the number of traumatic experiences across life. ^e^PSQ: Prenatal Stress Questionnaire assesses adverse events experienced by the clients' mother during pregnancy. Results are presented in the format M ± SD, score ranges are added in brackets.

*Stress measures correlated *with each other, suggesting accumulating or interacting effects of stress: In both groups, prenatal stress correlated with stress load across life periods, and in the patient group, traumatic experiences (PDS) correlated with stress load across life (see Table 3).

**Table 3:** Relationship between Measures of Stress Load

	**ELS-events/** **ELS-score**	**PPS-events/** **PPS-score**	**AS-events/** **AS-events**
**PSQ **(prenatal stress)			
patients:	*r*_*s *_= .53***/.51***	*r*_*s *_= .53***/.56***	*r*_*s *_= .20*/.30**
comparison group:	*r*_*s *_= .37*/.45*	*r*_*s *_= .54**/.69***	*r*_*s *_= .36*

**PDS **(traumatic stress)			
patients:	*r*_*s *_= .42***/.39***	*r*_*s *_= .57***/.48***/	*r*_*s *_= .31**/.27*
comparison group:	n.s.	n.s.	n.s.

*Note*. ^a ^ELS: Early life stress before the age of 6 years. ^b^PPS: prepubertal stress before the individual onset of puberty. ^c^AS: Adulthood stress between puberty and the current age. PSQ: Prenatal Stress Questionnaire, PDS: Posttraumatic Stress Diagnostic Scale see note of Table 2. Correlations are represented by Spearman's rho. * *p *< .05, ** *p *< .01, *** *p *< .001.

A comparison of the four *stress domains *(trauma, emotional neglect, physical punishment, and sexual abuse) disclosed emotional neglect as dominant experience across groups and life periods (Group × Stress domain: ELS: F(3,372) = 3.23, *p *< .05; PPS: F(3,372) = 6.64, *p *< .001; AS: F(3,372) = 6.65, *p *< .001; main effects Stress domain and Group, p < .001 for all life periods). In addition, patients reported more violence in their families during childhood and adolescence than comparison subjects (F(1,125) = 22.00, *p *< .001); and patients were more often separated from their biological mother for a time period of least 3 month before puberty (21% of the patient sample, no comparison subject, chi^2 ^= 7.76, p < .01). When subjects were asked to evaluate their childhood according to school grades (between 1 = best and 6 = miserable), patients assigned less favorable grades to their childhood (3.7 ± 1.7) than comparison subjects (2.4 ± 1.2; F(1,125) = 14.84, *p *< .001).

Stress load differed between the *diagnostic groups*: As evident from Table 4 and 5, patients with personality disorders were characterized by the highest childhood and prepubertal stress load on all measures including traumatic events (PDS) and prenatal stress load (PSQ). Prepubertal stress was also higher in MDD than in schizophrenia patients and drug addicts. A predominance of emotional and sexual abuse in PD patients relative to the other diagnostic subgroups was further confirmed by the interactions Diagnostic group × Stress domain (ELS: F(9, 276) = 2.8, p < .01; PPS: F(9,276) = 3.45, p < .01). The different distribution of stress load in diagnostic groups was confirmed, when stress-related subgroups were assigned to a 'high stress' and a 'low stress' group (see methods). Across life periods the 'high-stress' group included more MDD and PD patients than the 'low stress' group, while a higher proportion of schizophrenic patients were assigned to the 'low stress' groups and a higher proportion of drug abuse patients to the 'low ELS' group (see Table 6).

**Table 4:** Stress Scores for the Diagnostic Subgroups and Periods of Life

**Diagnostic Subgroup**	**ELS ** **Events** **Stress load**	**PPS ** **Events ** **Stress load**	**AS ** **Events** **Stress load**	**PDS ** **Events**	**PSQ ** **Events**
**Major Depressive Dis. **(N = 39)	7.3 ± 5.6	12.1 ± 6.7	5.4 ± 3.9	2.44 ± 1.8	4.13 ± 3.7
	51.5 ± 61.1	194.1 ± 175.6	161.3 ± 188.9		
**Schizophrenia **(N = 32)	3.5 ± 4.1	7.6 ± 5.6	3.9 ± 2.8	1.06 ± 1.5	2.84 ± 2.5
	29.9 ± 57.6	100.0 ± 136.0	91.0 ± 115.0		
**Drug Abuse **(N = 15)	3.2 ± 3.8	10.0 ± 6.1	5.9 ± 4.1	2.8 ± 1.9	2.8 ± 2.7
	23.1 ± 36.4	140.1 ± 108.2	155.7 ± 153.1		
**Personality Disorder **(N = 10)	12.1 ± 7.6	20.2 ± 7.3	5.7 ± 3.5	3.8 ± 0.9	6.5 ± 4.2
	122.8 ± 121.0	366.4 ± 230.5	149.9 ± 113.7		
**Subgroup differences:**	9.32***	10.13***	1.58, n.s.	8.81***	3.84**
F(3,91) =	5.91**	7.43***	1.35, n.s.		

*Note*: See note in Table 2: Data represent the cumulated number of experienced events (top rows) and the stress load calculated accordingly to the ETI guidelines (bottom row). ELS: Early life stress before the age of 6 years. PPS: Prepubertal stress before the individual onset of puberty. AS: Adulthood stress between puberty and the current age. PDS: number of traumatic experiences across life. PSQ: Prenatal Stress Questionnaire; see note in Table 2. Results are presented in the format M ± SD. Statistical significance: *: p < .05, **: p < .01, ***: p < .001.

**Table 5:** Posthoc Statistical Verification of Diagnostic Group Differences in Stress Load

	**ELS**	**PPS**	**AS**	**PDS**	**PSQ**
**Major Depessive Dis.**					
> Comparison Ss	t(68) = 3.8**	t(68) = 4.5***	t(68) = 3.3**	t(68) = 5.0***	t(68) = 3.6***
> Schizophrenia		t(69) = 2.5*	t(69) = 1.8^t^	t(69) = 3.4**	t(69) = 1.7t
**Schizophrenia**					
> comparison Ss	t(61) = 1.8^t^	t(61) = 2.0*	t(61) = 2.0*	t(45) = 3.3**	t(61) = 2.3*
**Drug Abuse**					
> Comparison Ss		t(44) = 3.7***	t(44) = 3.7***	t(44) = 5.1***	t(44) = 1.9t
**Personality Disorder**					
> MDD	t(47) = 2.7**	t(47) = 2.6*		t(47) = 2.3*	t(47) = 1.8^t^
> Schizophrenia	t(40) = 3.0**	t(40) = 4.5***		t(40) = 5.4***	t(40) = 3.4**
> Drug Abuse	t(23) = 5.1***	t(23) = 3.3**			t(23) = 2.7*
> Comparison Ss	t(39) = 3.8**	t(39) = 7.1***	t(39) = 4.1***	t(39) = 9.5***	t(39) = 5.3***

Note: ELS: ETI-score before th age of 6 years, PPS: ETI-score between age of 6 years and onset of puberty, AS: ETI-score between puberty and current age, PDS: number traumatic events across life; PSQ: stress load of client's mother during pregnancy. Statistical Significance: t: p < .1*: p < .05, **: p < .01, ***:p < .001

**Table 6:** Assignment of the patients with Different Diagnoses to High and Low Stress Groups separately for the Early Life -, Prepubertal -, and Adulthood Stress Load

	**Stress ** **Group**	**ELS ** **Number of ** **patients**	**PPS ** **Number of** **patients**	**AS ** **Number of ** **patients**
**Major Depressive Dis.**	**high**	14	19	18
	**low**	11	10	11
**Schizophrenia**	**high**	5	7	6
	**low**	18	18	15
**Drug Abuse**	**high**	2	5	6
	**low**	8	4	4
**Personality Disorder**	**high**	7	8	3
	**low**	3	0	1
**Group differences: **chi^2 ^(3)		11.14, *p *< .001	15.20, *p *< .01	6.64, *p *< .01

*Note*. Number of patients assigned to the 'high' and 'low stress' group separately for ELS, PPS, and AS. High stress load: stress-scores exceeding 2 SD above the mean of the comparison group. Low stress load: stress-scores below the mean of the comparison group. ELS: Early life stress; PPS. Prepubertal stress, AS: Adulthood stress (see note in Tables 2 and 4).

The relationship between *current distress or measures of psychopathology *and early life stress was further investigated by relating measures of stress load to overall severity of disorder, to affective symptoms (PANAS) and BDI), and to posttraumatic stress symptoms (intrusion, avoidance, and arousal according to the PDS). As summarized in Table 7 early life stress (ELS) in patients varied with more severe general psychopathology (BPRS), while no relationships were found between number of hospitalizations, GAF, or current drug abuse. Across subjects, higher stress load before puberty was related to more negative affect estimated for the week before the interview, while no relationship was found for positive affect. In patients, early life varied with more pronounced depression (BDI). Comparing 'stress groups' confirmed significantly more pronounced affective symptoms in the 'high-' compared to the 'low-stress' patient groups (see Table 7). In schizophrenia patients, hallucinations were related only to adult stress load (BPRS Item: *r*_*s *_= .30, p = .09, hallucinations and delusions subscores of the PANSS-P: *r*_*s *_= .37, p < .05), particularly to emotional (*r*_*s *_= .38, p < .05) and sexual abuse (*r*_*s *_= .31, p = .06).

**Table 7:** Relationship between Stress Load and Measures of Psychopathology

	**ELS-events/** **ELS-scores**	**PPS-events/** **PPS-scores**	**AS-events/** **AS-scores**
**BPRS**			
patients:	*r*_*s *_= .22*/.21*	n.s.	n.s.

**PANAS-negative affect**			
patients:	*r*_*s *_= .40***/.39***	*r*_*s *_= .39***/.37***	*r*_*s *_= n.s./.23*
comparison group:	*r*_*s *_= .36*/n.s.	n.s.	n.s.
total sample:	*r*_*s *_= .46 ***/.47***	*r*_*s *_= .46***/.48***	*r*_*s *_= n.s./.36***

high- vs. low-stress groups	N = 28 vs. N = 40	N = 39 vs. N = 32	
	t(66) = 3.80 **	t(69) = 3.40**	

**BDI**			
patients:	*r*_*s *_= .35***/.33**	*r*_*s *_= .29**/.30**	*r*_*s *_= n.s. ?/.22*
comparison group:	n.s.	n.s.	n.s.

high- vs. low-stress groups	N = 28 vs. N = 39	N = 39 vs. N = 32	
	t(65) = 3.16**	t(69) = 3.33**	

**PTSD symptoms (PDS)**			
patients:	*r*_*s *_=.46***/.50***	*r*_*s *_=.47***/.50***	*r*_*s *_=.32***/.42**
comparison group:	n.s.	n.s.	n.s.

high- vs. low-stress groups	N = 28 vs. N = 40	N = 39 vs. N = 32	N = 32 vs. N = 31
	t(66) = 5.39***	t(69) = 5.42***	t(61) = 3.58***

Note: BPRS: Brief Psychiatric Rating Scale. PANAS: Positive and Negative Affect Scale. BDI: Beck Depression Inventory. PTSD symptoms (PDS): severity (intrusions, avoidance, arousal) of the worst traumatic event according to Posttraumatic Stress Diagnostic Scale. Correlations are represented by Spearman's rho. Significance levels of correlation coefficients and subgroup comparisons: * *p *< .05, ** *p *< .01, *** *p *< .001.

**Figure 1:**
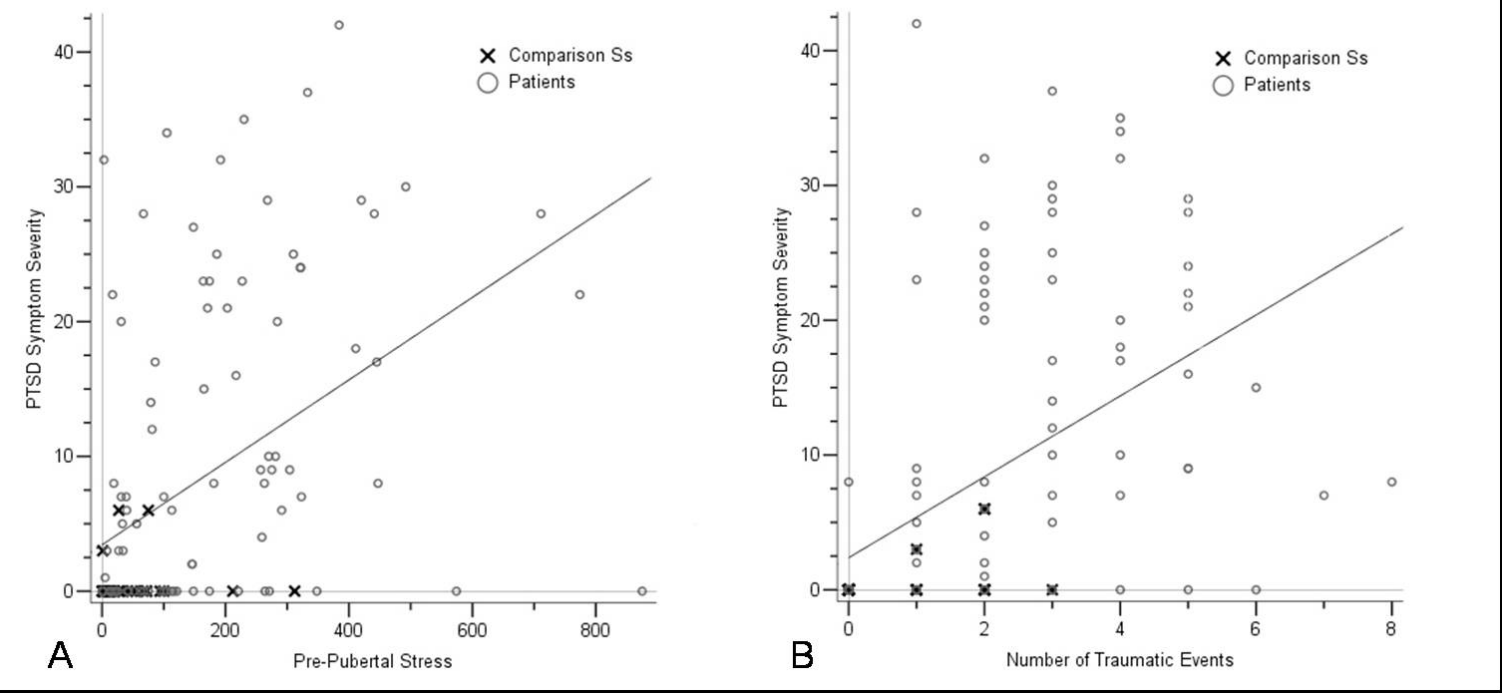
A: Scatterplot illustrating the relationship between the amount of prepubertal stress load (ETI-score, abscissa) and PTSD symptoms (ordinate) in patients (open circles) and comparison subjects (crosses). B: Scatterplot illustrating the relationship between the traumatic events (PDS number of traumatic events, abscissa) and PTSD symptoms (ordinate) in patients (open circles) and comparison subjects (crosses).

Severity of PTSD symptom severity was related to stress load in all life periods (see Table 7, Figure 1a). The PDS verified a PTSD-diagnosis in 28% of the patient group, while none of the comparison subjects met the diagnostic criteria. A significant relationship between the number of traumatic events and PTSD symptoms (Figure 1b) was confined to the patient sample (intrusions: *r*_*s *_= .51, avoidance: *r*_*s *_= .77, and hyperarousal: *r*_*s *_= .77, all *p *< .001). In the comparison group a correlation was only found for hyperarousal (*r*_*s *_= .39, *p *< .05). When PTSD symptoms were examined in those 24 patients and 19 comparison subjects, who did not report traumatic events in the PDS, those 12 patients, who experienced PTSD symptoms related to the most stressful ETI-item exhibited higher stress load than the 16 subjects without traumatic events and PTSD symptoms (ELS: F(1,26) = 4.96, *p *< .05; PPS: F(1,26) = 9.93, *p *< .01; AS: F(1,26) = 6.25, *p *< .05). This indicates that PTSD symptoms occur also as a consequence of severe non-traumatic stress load.

Applying linear regression models, a comorbid PTSD diagnosis was best predicted (for the total sample *R*^2 ^= .37, for the patient group *R*^2 ^= .32) by emotional neglect before the age of 6 years (total sample: *r*_*s *_= .48, β = .44, *p *< .001; patient group: *r*_*s *_= .49, β = .44, *p *< .001), sexual abuse between puberty and the age of 18 years (total sample: *r*_*s *_= .37, β = .16, *p *< .05; patient group: *r*_*s *_= .40, β = .16, *p *= .07), and the number of traumatic events (PDS; total sample: *r*_*s *_= .48, β = .23, *p *< .01; patient group: *r*_*s *_= .45, β = .21, *p *< .05).

## Discussion

Stress load in early childhood and before the onset of puberty, but not in adulthood, were more prominent in adult psychiatric patients than in non-psychiatric comparison subjects. This is in line with earlier assumptions of a relationship between childhood adverse experiences and psychopathology. This relationship by itself does not demonstrate causality. Although a genetic contribution to severe mental disorders is not disputed, a genetic contribution of perhaps at most 50% implies that disorders cannot be reduced to genetic bases alone. It is tempting to attribute a proportion of the remaining variance to individual history including stressful experiences. However, it cannot be concluded from a relationship such as that found in the present study whether the vulnerability for a mental disorder influenced the vulnerability for childhood adverse experience or vice versa, or whether a third factor contributed to both risks. Although models of genotype-environment interaction may help to understand interindividual variation in phenotypes, the striking heterogeneity of symptoms and psychopathology in most of the major mental disorders continues to challenge etiological models, diagnostics and treatment. The concept of allostatic load [49,50] may be helpful in the investigation of a potential environmental share to the biosocial co-constructivism in mental disorders and in the understanding of a distinct subset (endophenotype) of mental disorder.

The present results support earlier assumptions of childhood as critical developmental period. Several explanations for the impact of early life stress on later psychopathology are discussed: Stress-related enhancement of CRF secretion during 'sensitive periods' of brain plasticity in childhood and adolescence [11,23,51] may prompt hippocampal volume loss, or sensitize and alter feedback properties of the HPA axis. Stress-induced hyperactivity of the stress system operates, by way of combined effects of CRF and glucocorticoids, to drive plastic changes in amygdala, hippocampus, and the mesocorticolimbic dopaminergic system [8]. While no conclusion regarding one another explanation can be drawn from the present results, the prominence of childhood stress load and its relationship with prenatal stress strengthens the assumption that early life stress plays a significant role as vulnerability factor, vulnerability considered as function of genetic and neurodevelopmental pathology of brain systems that are also related to stress systems.

The present study included patients with different diagnoses in order to explore, whether stress load would exert its impact on psychopathology beyond diagnostic boundaries. Results disclosed pronounced differences in stress load between diagnostic groups. This may be considered a confounding variable, making it difficult to specify the impact of early life stress. It may also be considered an indication of the bio-social co-constructivism [52]. Relationships between stress load and other vulnerability factors may vary between disorders: For MDD, present results of a relationship between early life stress and adult psychopathology confirm previous reports [10]. For schizophrenia patients, present results did not support a relationship between childhood trauma and psychotic symptoms [15,30,32]. This may be explained in part by a small and selected sample, in which early life stress was lower than expected, potentially preventing a clear relationship to psychopathology. Still, psychotic symptoms such as hallucinations and delusions were related to more recent stress load in adulthood, suggesting an interaction between disorder and stress sensitivity. In drug addicts, a relationship between stress load on craving [53] and an association between PTSD, but not trauma only [21] has been found. Although a comorbid diagnosis of personality disorder in 40% of the present sample of drug abuse patients might suggests severe impairment, the lack of comorbid PTSD diagnoses and low depression (BDI) in the present inpatients, who were beyond the withdrawal period, may in part account for the low stress load in this subgroup. Childhood stress load was most pronounced in patients treated for personality disorders. While this result is in line with other reports [e.g. [19,20]], it has to be verified for a larger sample.

The uneven distribution of stress load within diagnostic subgroups did not allow the comparison of stress-related subgroups within each diagnostic category. Therefore, we can only speculate that early life stress interacts with disorder-specific factors, adding to biological vulnerability or reducing capacity for coping, which might add to the difficulty of coping with later symptoms or psychopathology. It remains to be clarified in further studies with larger samples balanced for diagnosis and stress load, whether a stress-related subgroup of mentally ill individuals across diagnostic boundaries can be identified.

Affective psychopathology and PTSD symptoms were strongly related to early life stress. This supports previous results and the assumption of a mediating function of affect [29]. However, the relationship between psychotic symptoms and stress load in the schizophrenia sample also suggests considering a broader spectrum of functions and coping capacity in further studies.

A 'dose' or 'building block' effect has been described for PTSD [36] and psychotic symptoms [37]: with increasing number of traumata or more severe traumata, the probability to suffer from symptoms and a PTSD or the severity of psychotic symptoms increases. The present study showed that such a 'building block' effect also characterizes the relationship between the amount of non-traumatic adverse experiences and the severity of psychopathology. This suggests that early life stress may contribute to an increased sensitivity for psychological stress responses, including PTSD symptoms.

Methodological limitations of the present study hamper the interpretation of the results. Since the assumption of a similar distribution of stress load across diagnostic subgroups had to be rejected, the patient sample was split up into diagnostic subgroups of unequal size. Even the supposedly larger sample of almost 100 patients did not compensate for the unexpectedly pronounced differences in stress load between diagnostic subgroups, which prevented the comparison of stress-related (high vs. low) subgroups within each diagnostic group. The identification of a stress-related phenotype among mentally ill individuals requires further studies with larger samples balanced for stress load and disorder. Another limitation of the present study resulted from the clinical setting, a Center for Psychiatry, which primarily treats chronic inpatients from the region. The unexpectedly low stress load in schizophrenia and in drug abuse patients may be attributed to the selected samples. Further studies should control for this potential influence. Moreover, clinical routine at the Center for Psychiatry Research did not allow examination of non-medicated patients or subgroups of patients with monotherapy. Finally, the present study concentrated on affective psychopathology and general symptom severity. Psychotic symptoms were only explored in schizophrenia patients, although a relationship between childhood trauma and hallucinations has been reported beyond diagnostic boundaries [31,38]. Further specification of the impact of childhood stress load on psychopathology must consider a broader spectrum of measures of psychopathology.

## Conclusion

Increased early life stress was confirmed for a larger sample of psychiatric inpatients treated for severe mental disorders. Present results support the hypotheses of stress-sensitive periods during development and show that a 'dose'-effect, a relationship between the amount of stressful experiences and severity of distress, is not restricted to traumatic experiences and to PTSD. Results also suggest that relationships between early life stress and psychopathology vary between disorders, which may result from an interaction of early life stress with other vulnerability factors.

## Abbreviations

AS: adulthood stress; BDI: Beck Depression Inventory; BPRS: Brief Psychiatric Rating Scale; ELS: early life stress; ETI: Early Trauma Inventory; GAF: General Assessment of Functioning; ICD 10: International Classification of Diseases and Related Health Problems, 10^th ^Revision; MDD: Major Depressive Disorder; MINI: Mini-International Neuropsychiatric Interview; NA: negative affect; PA: positive affect; PANAS: Positive and Negative Affect Scale; PANSS: Positive and Negative Symptom Scale; PD:; personality disorder; PDS: Posttraumatic Stress Diagnostic Scale; PPS: prepubertal stress; PTSD: posttraumatic stress disorder.

## Competing interests

The authors declare that they have no competing interests.

## Authors' contributions

KW set up the study, accomplished data collection and analyses together with JB, BA and TP, and prepared the manuscript together with BR. BR designed the study protocol, supervised the study and wrote the manuscript together with KW. KH, KS, HW and KP were responsible for recruitment and diagnostic of patients and supervised the data acquisition as psychiatrists.

## Pre-publication history

The pre-publication history for this paper can be accessed here:


